# Body ownership and tactile processing: Effects of bilateral rubber hand illusion on tactile temporal order judgment

**DOI:** 10.1177/20416695241308146

**Published:** 2024-12-23

**Authors:** Satoshi Shibuya, Yukari Ohki

**Affiliations:** Department of Integrative Physiology, School of Medicine, Kyorin University, Tokyo, Japan; Department of Integrative Physiology, School of Medicine, Kyorin University, Tokyo, Japan

**Keywords:** hand ownership, temporal order judgment, rubber hand illusion, multisensory integration

## Abstract

In the rubber hand illusion (RHI), individuals perceive a fake hand as their own if an unseen hand and a visible fake hand are stroked simultaneously. We examined how the RHI on either hand influenced the temporal order judgment (TOJ) of bimanual stimulation. In Experiment 1, participants performed TOJ during RHI or non-RHI conditions. When the fake hand was oriented in an anatomically incongruent position (non-RHI condition), the point of subjective simultaneity (PSS) from TOJ showed no difference between the stroke sides. However, during the RHI condition, the PSS tended to shift in the opposite direction to the stroke, resulting in a significant difference between the sides. This implies slower tactile processing in the illusion-affected hand. When participants performed an identical TOJ while watching the fake hand without stroking (Experiment 2), no PSS modulation was found. Our findings suggest the possibility that the RHI attenuates tactile processing, but its magnitude is small.

The perception that one's body belongs to oneself is referred to as the sense of body ownership, which represents a fundamental aspect of self-consciousness (Gallagher, 2000). Most studies have investigated body (hand) ownership using the rubber hand illusion (RHI) (Botvinick & Cohen, 1998) in which participants perceive a fake hand as their own if an unseen real hand and a visible fake hand are stroked synchronously with paint brushes (i.e., visuo-tactile stimulation). However, placing the fake hand in an anatomically implausible position (e.g., the fake hand rotated by 90° relative to the real hand) reduces the illusion (Pavani et al., 2000; Tsakiris & Haggard, 2005). This implies that the RHI is sensitive to preexisting representations of one's own body (i.e., body representation). In addition, asynchronous stroking of the real and fake hands (discrepancy > 0.3 s) degrades the illusion (Shibuya et al., 2019; Shimada et al., 2009). This suggests that multisensory temporal integration of visual and tactile inputs is important to induce the visuo-tactile RHI, although mere observation of the fake or virtual hand induces illusory hand ownership (Casula et al., 2022; Pavani et al., 2000; Tieri et al., 2015). Therefore, these spatial and temporal inconsistencies are frequently used as control conditions in RHI studies (Kalckert & Ehrsson, 2012; Tsakiris & Haggard, 2005; Tsakiris et al., 2007).

Although synchronous visuo-tactile stimuli are delivered using brushstrokes and vibrators (Kanayama et al., 2007; Pavani et al., 2000) during RHI induction, multisensory conflicts would occur because the spatial positions at which the real and fake hands are stimulated are different. Thus, it is intriguing how the brain resolves conflicts to integrate visual and tactile inputs during the RHI. Within the framework of predictive coding, the brain continually interprets sensory information using a hierarchical generative model of the world. Any difference between the predicted and actual sensory data at any given level generates prediction errors that are passed on to the higher level via bottom-up (forward) connections. To minimize prediction errors, the brain adjusts top-down predictions based on the internal model to explain prediction errors at lower levels (Limanowski & Blankenburg, 2015; Murray et al., 2002). According to predictive coding, the RHI is caused by minimizing the prediction errors that arise from visual (i.e., seeing the touch) and tactile inputs (i.e., feeling the touch). One effect of the brain's strategy to minimize errors is the attenuation of somatosensory processing in the real hand (Zeller et al., 2015). This notion is supported by earlier psychophysical studies using bimanual temporal order judgment (TOJ) (Moseley et al., 2008) and cross-modal congruency task (CCT) (Walton & Spence, 2004). In the TOJ study, right-handed participants received two tactile stimuli delivered to each hand with a certain stimulus onset asynchrony and judged their temporal order. Using a combination of the RHI and TOJ, it was found that in the synchronous stroking (RHI) condition, the point of subjective simultaneity (PSS) obtained from the TOJ shifted as tactile processing of the stroked right hand (i.e., illusion-affected hand) was slower than that of the nonstroked left hand (i.e., nonaffected hand), but not in the asynchronous stroking (non-RHI) condition. Our interpretation of this phenomenon suggests that the attenuation of somatosensory processing in the RHI-affected hand leads to a prioritization of tactile inputs from the nonaffected hand over those from the illusion-affected hand in the tactile TOJ. In the CCT study (i.e., visuo-tactile interference task), the visual capture of tactile sensation induced by the rubber hands decreases the effect of tactile distractors on visual discrimination, suggesting a reduced subjective feeling of tactile stimulation (Walton & Spence, 2004). In related tactile TOJ studies on patients suffering from complex regional pain syndrome (CRPS) of one arm (i.e., either right- or left-sided symptoms), PSS values showed that tactile processing of the pain-affected arm was slower than that of the unaffected arm (Moseley et al., 2009; Reid et al., 2016; see also Moseley et al., 2012).

In a study by Moseley et al. (2008), only the right hand (i.e., the dominant hand) was used as the RHI-affected hand. Given the slower tactile processing of the pain-affected arm, regardless of which arm is affected (Moseley et al., 2009; Reid et al., 2016), it is predicted that an ownership-dependent PSS shift occurs even if the nondominant (left) hand is used as the illusion-affected hand. However, handedness reportedly modulates the RHI (Dempsey-Jones & Kritikos, 2019) and tactile TOJ (Wada et al., 2004). For the RHI, proprioceptive drift, which measures the RHI by assessing the shift in the proprioceptively perceived position of the hand toward the fake hand after the illusion, was greater in the nondominant hand than in the dominant hand (Dempsey-Jones & Kritikos, 2019). In the tactile TOJ, it was observed that some right handers experienced a delay in perceiving stimuli on their left (nondominant) hand compared to their right (dominant) hand (Wada et al., 2004). In addition, the right hemisphere is dominant for spatial processing in the right hander compared to the left hander (Vogel et al., 2003). Therefore, we hypothesized that greater effects of the RHI on tactile TOJ may be observed for the nondominant (left) than for the dominant (right) hand in the right-handed participants. In the current study, Experiment 1 extends the evidence of Moseley et al. (2008) from two perspectives. Using the RHI and tactile TOJ, we first tested whether ownership-dependent PSS modulation occurred in the same or different way in both hands. A comparison between the dominant (right) and nondominant (left) hands in ownership-dependent PSS modulation may offer beneficial insights regarding the neurocognitive processing underlying this phenomenon. Second, while temporal incongruency (i.e., asynchronous stroking) was used as the control (non-RHI) condition in the study by Moseley et al., (2008), we used spatial incongruency (i.e., fake hand placed in anatomically implausible position) in the non-RHI condition; synchronous stroking was delivered in both the RHI and non-RHI conditions in our study. The positive results of this study would provide stronger evidence that PSS changes during the RHI are caused by illusory hand ownership rather than mere synchronous visuo-tactile stroking. In Experiment 2, we required the participants to conduct an identical TOJ while watching the fake hand to eliminate the possibility that the PSS modulation was due to the mere visual information of the fake hand.

## Methods

### Participants

We selected 18 participants for Experiment 1 (11 men and 7 women; 22.4 ± 2.5 years old, mean ± SD) and 20 participants for Experiment 2 (10 men and 10 women; 25.7 ± 7.4 years old). The authors were not included as participants in this study. All participants were right-handed according to the Edinburgh Handedness Inventory (Oldfield, 1971). This study was approved by the Institutional Review Board of the Kyorin University School of Medicine and conducted according to the principles and guidelines of the Declaration of Helsinki. All participants provided written informed consent before participating in the study. The number of participants required for each experiment was based on the previous study (*n* = 15; Moseley et al., 2008). We added 20–33% (*n* = 3–5) to the number of participants in the previous study to consider those with a poor fit to the psychometric function of the tactile TOJ.

### Procedures

**Figure 1.**
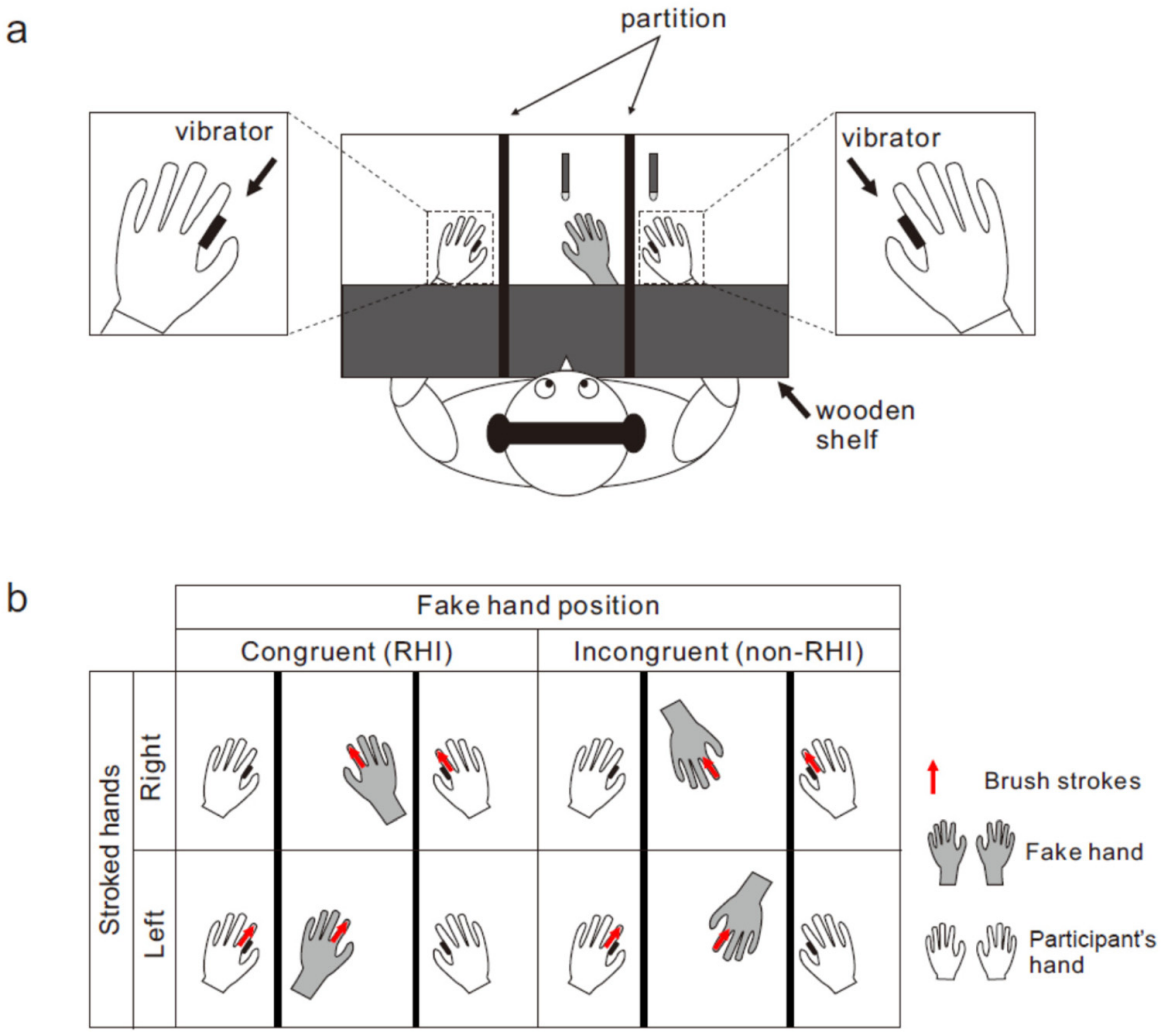
Experimental setup of experiment 1. (a) Participants sat with their both hands palm down on a desk (top view). For inducing a rubber hand illusion (RHI), an experimenter stroked the index fingers of the fake hand and the corresponding participant's (right or left) hand twice synchronously at approximately 0.5 Hz using two paintbrushes. Synchronous stroking was followed by the delivery of two rapid vibrotactile stimuli to both hands (one to each hand) for temporal order judgment (TOJ). Participants reported the temporal order of two stimuli. (b) Each participant completed four experimental conditions with a 2 × 2 factorial design: *fake hand position* (congruent [RHI] vs. incongruent position [non-RHI]) and *stroked hands* (right vs. left hand). Red arrows denote the locations and directions of visuo-tactile stroking.

The participants sat at a desk with both hands palm down (Figure 1a). A wooden shelf was placed approximately 16 cm above each participant's forearm. A life-sized fake right or left hand was placed in front of each participant. The distance between the index fingertips of the real and fake hands was maintained at 20 cm. The space between the wooden shelf and the participants’ torsos was concealed by a black cape (not shown in Figure 1a). Two partitions were placed between the fake hand and the participants’ hands to prevent them from viewing their hands. Solenoid skin contactors applied a brief vibrotactile stimulation (duration: 10 ms) to the medial surfaces of the proximal phalanges of both index fingers of the participant's hand. Two foot pedals were positioned beneath the participants’ toes to judge the temporal order of the vibrotactile stimulation. In the tactile TOJ task, two successive vibrotactile stimuli were delivered, one to each finger, with stimulus onset asynchrony (SOA). The SOA was randomly assigned for each trial from 8 values (–200, −100, −50, −25, 25, 50, 100, 200 ms) in Experiment 1 and 10 values (–240, −120, −60, −30, −15, 15, 30, 60, 120, 240 ms) in Experiment 2. Positive values indicate that the right finger was stimulated earlier than the left finger, and vice versa. White noise was played through headphones placed over the participants’ plugged ears to mask the stimulator's sounds.

In both experiments, each participant completed four experimental conditions with a 2 × 2 factorial design: *fake hand position* (congruent vs. incongruent position) and *stroked hands* (Experiment 1: Figure 1b) or *observed fake hand* (Experiment 2) (right vs. left hand). Although the position of the fake hand was congruent with that of the participant's stroked hand in the congruent condition, it was rotated 180° relative to the participant's hand in the incongruent condition. The order of the four conditions was randomized across participants. In Experiment 1, the experimenter stroked the index fingers of the fake hand and the corresponding participant's hand twice synchronously at approximately 0.5 Hz using two paint brushes (red arrows in Figure 1b). Synchronous stroking was followed by the delivery of rapid vibrotactile stimulation to both hands. Participants judged which hand was stimulated later by pressing the right or left foot pedals. The participants were given as much time as needed, but no feedback was given on whether their judgments were correct. During the experiment, the participants focused on the index fingertip of the fake hand. In Experiment 1, the combination of two strokes and one TOJ was repeated 80 times (8 SOAs × 10 trials) for each condition. The procedure of Experiment 2 was identical to that of Experiment 1, except that no stroking was performed. The participants performed the TOJ while watching the fake hand. Participants in Experiment 2 performed 100 trials (10 SOAs × 10 trials) for each condition.

In Experiment 1, after completing each condition, the participants reported their subjective feelings during the experiment using a questionnaire. The questionnaire comprised four items. Q1: “*It seemed as if I were feeling the touch of the paintbrush in the location where I saw the rubber hand touched*.” Q2: “*I felt as if the rubber hand were my hand.”* Q3: *“It appeared (visually) as if the rubber hand were drifting toward my hand.”* Q4: “*It seemed as if I might have more than one left (right) hand or arm.”* Participants responded to all items using a 7-point Likert scale ranging from +3 (strongly agree) to −3 (strongly disagree). These items are classified into two categories: *ownership* (Q1 and Q2) and *ownership control* (Q3 and Q4).

## Results

**Figure 2.**
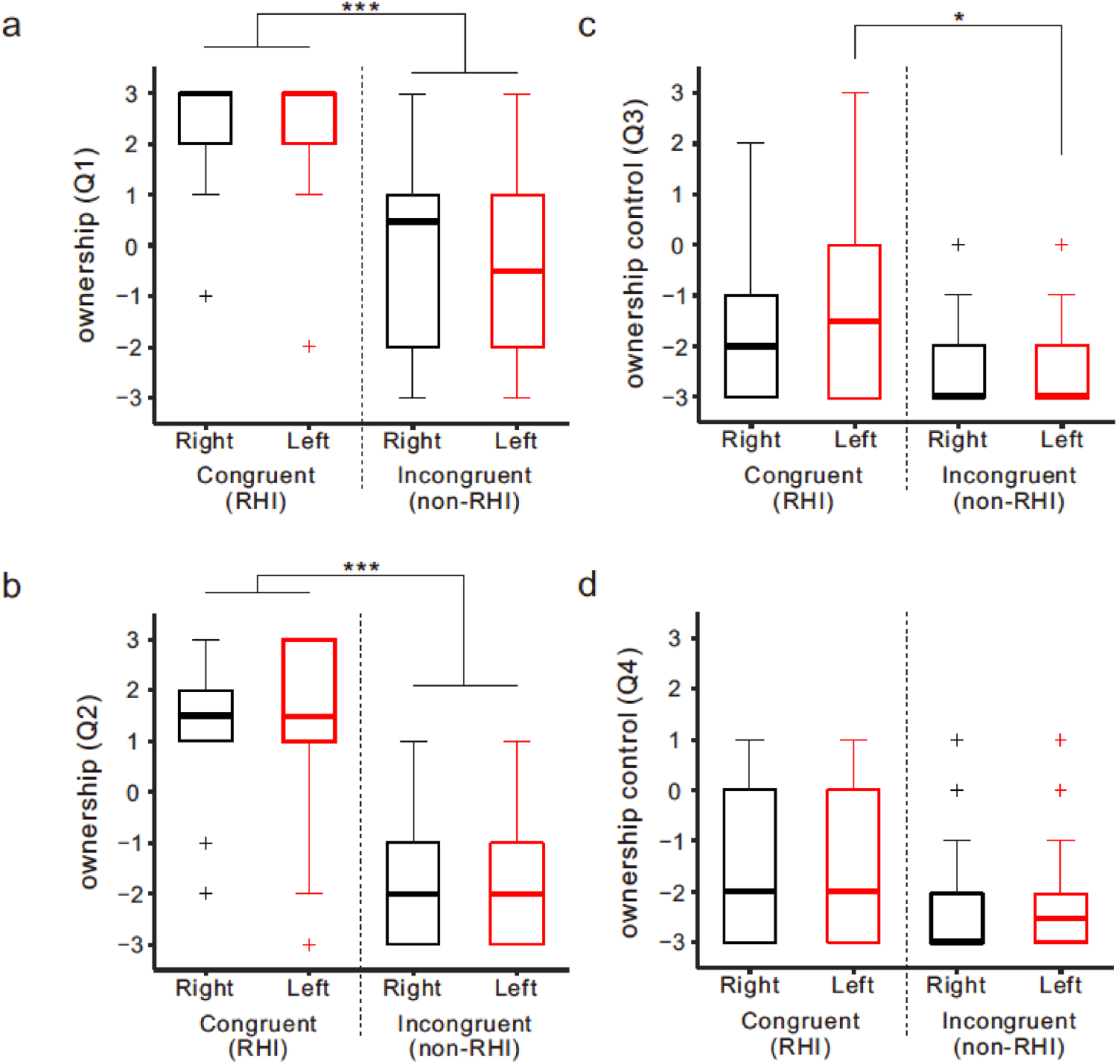
Questionnaire ratings of the congruent (RHI; left) and incongruent conditions (non-RHI; right) in experiment 1 are presented against the stroked right (black) and left hands (red) for the *ownership* (Q1 [a] and Q2 [b]) and *ownership control* (Q3 [c] and Q4 [d]). Boxes and thick lines denote the interquartile ranges (IQRs) and medians, respectively. Small plus indicates outlier participants. Whiskers represent either additional data points or extend to 1.5 × IQR. Significance is donated using asterisks (***p < .001; *p < .05).

Figure 2 shows the median and interquartile ranges of the questionnaire ratings in Experiment 1. The null hypothesis of equal medians across the four conditions was rejected in Q1 (a), Q2 (b), and Q3 (c) (χ^2^[3] > 17.5, *ps *< .001, all; Friedman test) but not in Q4 (d) (*p *> .3). In both Q1 and Q2 (*ownership*), the medians of the congruent (RHI) conditions were beyond +1 (i.e., affirmation) and statistically larger than those of the incongruent (non-RHI) conditions, regardless of the hand stroked, right or left (*ps *< .001; Bonferroni test). In the congruent condition, no difference between the right (black) and left (red) fake hands was found in Q1 and Q2 (*ps *= 1.0, for both). Although the ratings of Q3 (*ownership control*) were low overall (medians < −1, all), the median (−1.5) of the congruent condition in the left hand was higher than that of the incongruent condition (−3) (*p *< .05).

**Figure 3.**
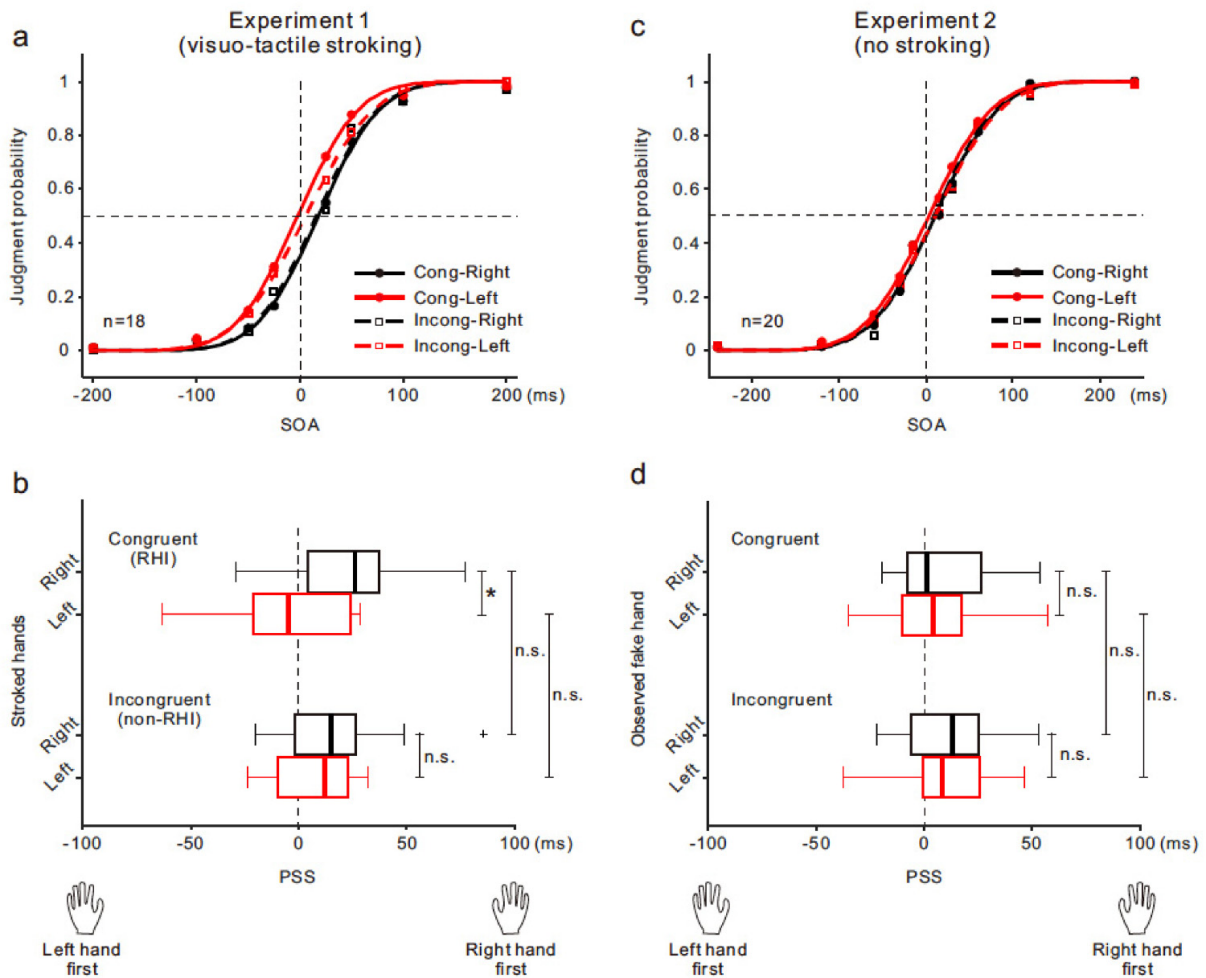
(a, c) The mean judgment probability that the right hand was stimulated earlier than the left hand is plotted against the stimulus onset asynchronies (SOA) in experiments 1 (*n* = 18; a) and 2 (*n* = 20; c). Positive SOA indicates that the right hand was stimulated earlier than the left hand. Solid (congruent condition) and dashed (incongruent condition) curves are obtained by fitting a cumulative Gaussian function to the judgment probability as a function of SOA. Colors of curves and symbols show the stroked hands (Experiment 1) and observed fake hand (Experiment 2) (i.e., black [right hand] and red [left hand]). (b, d) Boxplots of the point of subjective simultaneity (PSS) of the congruent (top) and incongruent conditions (bottom) are shown for each stroked hand (Experiment 1; b) and observed fake hand (Experiment 2; d). Boxes and thick lines donate the interquartile ranges (IQRs) and medians, respectively. A small plus indicates an outlier subject. Whiskers represent either additional data points or extend to 1.5 × IQR. Significance is donated using an asterisk (*p < .05).

Figure 3 shows the TOJ results for Experiments 1 (a, b) and 2 (c, d). For each condition, we first computed the order-judgment probability of the right finger being stimulated earlier in each SOA. Subsequently, we determined the PSS, where the probability was .5, by fitting a cumulative Gaussian function to the judgment probability as a function of the SOA. In Experiment 1, the probability was approximated well by the Gaussian function under all conditions when the data were pooled across all participants (a). Friedman test for PSS values obtained from all participants (b) indicated that the null hypothesis of equal medians across the four conditions was rejected (χ^2^[3] = 11.5, *p *< .01). In the congruent (RHI) condition, the difference between the stroked right (black) and left (red) hand conditions was statistically significant (right hand vs. left hand: 26.1 vs. −4.8 ms [median]; *p *< .05; Bonferroni test) but not in the incongruent (non-RHI) condition (14.7 vs. 11.8 ms; *p *= 1.0). While comparisons between the congruent and incongruent conditions in each stroked hand showed no statistically significant differences (*ps *= 1.0, for both), the difference between the two conditions tended to be larger in the left hand than in the right hand (11.4 vs. 16.6 ms). A similar curve fitting to the judgment probability (c) and statistical analyses of the PSS data (d) were performed in Experiment 2. The results demonstrated no difference in the PSS scores among the four conditions (*p *> 0.9, Friedman test). We performed a correlation analysis between *ownership* ratings (Q1 and Q2) and PSS values in Experiment 1. However, a significant correlation between the two in each hand was not identified (*r *= .07−.33; *ps *> .17, all; Pearson's product–moment correlation).

## Discussion

The questionnaire results of Experiment 1 confirmed that most participants perceived illusory ownership of the fake hand in the RHI (i.e., congruent) condition because the ratios of affirmative responses in *ownership* ratings were much higher in both the right (Q1:94% [17/18]; Q2:83% [15/18]) and left (Q1:94%; Q2:78% [14/18]) hands. Although significant differences in *ownership* ratings were found between the RHI and non-RHI (i.e., incongruent) conditions, there was no difference between the stroked (i.e., illusion-affected) right and left hands. This result is consistent with previous studies that found that stroked hands or handedness did not influence the strength of the RHI based on questionnaire ratings (Ocklenburg et al., 2011; Smit et al., 2017); however, these factors may modulate other physiological (Ocklenburg et al., 2011) and behavioral measures (Dempsey-Jones & Kritikos, 2019).

Regarding bimanual TOJ, the results of Experiment 1 showed a significant PSS difference between the stroked right and left hands in the RHI condition, but not in the non-RHI condition. Based on the PSS (median) of the non-RHI condition, this difference between both hands resulted from the PSS shifting in opposite directions to each other in the RHI condition (Figure 3b), partially supporting the idea that tactile processing of the stroked (i.e., illusion-affected) hand was slower than that of the nonstroked (i.e., nonaffected) hand. Moreover, the results of Experiment 2 (i.e., no stroking) eliminate the possibility that the PSS difference in Experiment 1 was caused by mere visual information from the fake hand. Our findings replicate and extend the evidence of Moseley et al. (2008) in that the difference in hand ownership, which was manipulated by changing the spatial positions of the synchronously stroked fake hand, modulates the PSS regardless of whether a dominant (right) or nondominant (left) hand is used as the RHI-affected hand. However, there are a few notable differences between previous studies and our study. First, a prior study found a significant PSS difference between the RHI (i.e., synchronous stroking) and non-RHI (i.e., asynchronous stroking) conditions in the single hand (i.e., right hand) (Moseley et al., 2008); however, such a PSS difference in each hand did not reach significance in our study. This shows that ownership-dependent PSS modulation in our study was small. Second, correlations between illusion strength and PSS values were observed in a previous study, whereas such correlations were not identified in our study. We propose two interpretations for these differences. One interpretation is that these differences were partially caused by PSS variability. The interparticipant PSS variability in our study (standard error of the mean [SEM]: 4.7−6.7 ms) was much higher than the previous one (1.2−2.2 ms). This may be due to methodological differences. For instance, the medial surfaces of the proximal phalanges of both index fingers were stimulated in our study, whereas the index fingertips (i.e., more sensitive areas) were stimulated in the previous study. Moreover, the participants in the previous study received a visual cue indicating that delivery of the vibrotactile stimuli was imminent. Another interpretation is that the effect of the RHI on the tactile TOJ is much weaker than we initially expected, based on the previous study (Moseley et al., 2008). Note that the sample sizes were relatively small in both the previous study (*n* = 15) and our study (*n* = 18; Experiment 1). While our findings partially support the argument that somatosensory (tactile) processing in the RHI-affected hand is attenuated, further research with a larger sample size is necessary to examine the robustness of ownership-dependent PSS modulation.

It is natural that the present results are associated with other psychophysical studies that demonstrated decreased tactile sensitivity during the RHI (Ataka et al., 2022; Rossi Sebastiano et al., 2022; see also Zopf et al., 2011). Based on predictive coding, Rossi Sebastiano et al. (2022) required participants to detect electrical (tactile) stimuli slightly above the perceptual threshold for both hands before and after the RHI of the right hand. The results showed that tactile detection was reduced in the illusion-affected right hand (i.e., increased tactile perceptual threshold) but not in the unaffected left hand. Similar results were also reported using the Semmes-Weinstein monofilament test as a clinical examination (Ataka et al., 2022), which was discussed based on its relevance to disownership (i.e., loss of own hand; Longo et al., 2008). Although our experiment delivered vibrotactile stimulation above the perceptual threshold, the decreased tactile sensitivity of the illusion-affected hand may be partially involved in ownership-dependent PSS modulation.

It is possible that the ownership-dependent PSS modulation is related to attentional processes during the RHI. For instance, spatial attention in the RHI condition may be focused on one (visual) location (i.e., the fake hand), whereas attention is divided across two (visual and tactile) locations (i.e., the real and fake hands) in the non-RHI condition (Rao & Kayser, 2017). In fact, if a visual stimulation related to a physical threat is presented in front of one or the other hand, greater tactile processing bias on the subsequent tactile TOJ is induced compared with neutral visual stimulation (Van Damme et al., 2009). Moseley et al. (2008) also argued that the ownership-dependent modulation of PSS resulted from a shift in attention toward the illusion-affected hand. According to predictive coding, attention plays a key role in the modulation of visual and tactile inputs when conflict occurs between the two modalities during RHI induction (Limanowski & Friston, 2020). If attention is related to the top-down modulation of sensory precision (i.e., reliability or uncertainty), an attentional shift toward the fake hand may influence visual and somatosensory processing of the real and fake hands during the RHI; this includes decreased reliability of somatosensory inputs from the real hand and increased reliability of visual inputs from the fake hand (Rossi Sebastiano et al., 2022). Sensory reliability also plays a crucial role in the Bayesian causal inference (BCI) model of the RHI (Chancel et al., 2022; Kilteni et al., 2015; Samad et al., 2015); the brain determines whether there is a common cause, such as the real hand, or two different causes, such as the real and fake hand, using available multisensory signals, including vision, touch, and proprioception. In support of the BCI model, studies have found that decreasing the reliability of visual (Chancel et al., 2022) or proprioceptive signals (Chancel & Ehrsson, 2023) enhances the strength of the visuo-tactile RHI. Indeed, attention modulates the spatial localization of visual and tactile stimuli according to the BCI principle (Badde et al., 2020). Moreover, the involvement of spatial (in)attention in the PSS bias has been suggested in a previous study of patients with CRPS (Moseley et al., 2009). In a study using the bimanual TOJ, PSS bias showed that the pain-affected arm's tactile processing was slower than that of the unaffected arm when the arms were not crossed. However, when the arms were crossed, the opposite was observed; the unaffected arm's tactile processing was slower, meaning that slowed tactile processing is associated with the space where the pain-affected arm normally resides (i.e., the default position) rather than the position of the affected arm itself. This pattern is consistent with data from patients with hemispatial neglect (inattention) (Bisiach & Luzzatti, 1978). Further studies are needed to clarify the relationship between ownership-dependent PSS modulation and attentional processing.

Which neural substrates are involved in ownership-dependent PSS modulation? There are two possible candidates. One is the primary somatosensory cortex (S1). Using electroencephalography (EEG) and the RHI, Zeller et al. (2015) found that somatosensory-evoked potentials (SEPs) via brush strokes (i.e., responses as early as 55 ms) were attenuated by illusory hand ownership. In a subsequent study, dynamic causal modeling of SEPs during the RHI showed that intrinsic connectivity in the S1 was attenuated during the RHI (Zeller et al., 2016). A recent EEG study with electrical stimulation of the median nerve also reported that the earliest components of the SEP (i.e., N1-P1 amplitude), which mostly originated from S1, were attenuated during the RHI (Sakamoto & Ifuku, 2021). Moreover, a transcranial magnetic stimulation (TMS) study examined the functional connections from the primary somatosensory and association cortices to the primary motor cortex (M1) and found that the RHI reduced somatosensory processing in the primary sensory and association cortices projecting to M1 (Isayama et al., 2019). Thus, it is assumed that processing in the S1 contributes to the ownership-dependent PSS modulation in this study. However, we must consider that some studies have shown negative findings regarding the attenuation of S1 activity during the RHI (Guterstam et al., 2019; Rao & Kayser, 2017). Using a well-controlled EEG experiment, Rao & Kayser (2017) observed lower amplitudes in event-related potentials related to the RHI at 120 ms over the right frontal region and at longer latencies (∼330 ms) over frontocentral areas, but no attenuation of the evoked responses was observed in the S1. A study using the RHI and electrocorticography (ECoG), which records cortical surface potentials, showed no illusion-dependent brain activity (high-γ power) in the S1 (Guterstam et al., 2019). Moreover, regarding the M1 activity, which is tightly linked to S1 activity (Shelchkova et al., 2023), previous studies have shown inconsistent findings. Some TMS studies reported a decrease in the amplitude of the motor-evoked potential during the RHI (della Gatta et al., 2016; Fossataro et al., 2018; Kilteni et al., 2016), whereas such attenuation was not supported in other TMS (Reader et al., 2023) and behavioral studies (Reader et al., 2021).

The other candidate is the posterior parietal cortex (PPC). Considering the results from patients with CRPS as described above (Moseley et al., 2009) and cross-hands deficit in the bimanual TOJ (Shore et al., 2002; Takahashi et al., 2013; Yamamoto & Kitazawa, 2001), it is possible that ownership-dependent PSS modulation is associated with multisensory processing in the spatial representations rather than processing in the somatotopical representation, like the S1. A functional magnetic resonance imaging (fMRI) study of the bimanual TOJ demonstrated that the inferior parietal region, part of the PPC, and the premotor cortex (PM) are involved in localizing tactile stimuli in the spatial map (Takahashi et al., 2013). Similarly, it is well known that the RHI involves activity in the PPC (specifically intraparietal area) and the PM (Ehrsson et al., 2004; Ehrsson 2020). PPC activity reflects not only the temporal integration of visuotactile stimuli (Chancel et al., 2022) but also the observed orientation of the fake hand during the RHI, as manipulated in the current study (Ehrsson et al. 2004). Based on this multisensory integration framework of body ownership (Ehrsson, 2020), the current results suggest that when referral of touch to the fake hand occurs owing to higher PPC activation during the RHI (see Figure 2a), it takes extra time to accurately localize vibrotactile stimulation to the illusion-affected hand in space for the subsequent TOJ task, compared to the nonaffected hand.

In our study, unlike the conventional RHI, we used two hands. This approach might be affected by an interaction between the multisensory representations of both hands during the RHI (Petkova & Ehrsson, 2009; Schaefer et al., 2013). Recently, Yamamoto (2024) showed that when both fake hands are on the table, synchronous visuotactile stimulation to one fake and one real hand can induce the embodiment of both fake hands, suggesting a unified representation of body ownership for both hands in the brain. Considering this, ownership of the nonstroked hand relative to the stroked hand might decrease (i.e., disownership), even though participants observed a single fake hand in our study. This could weaken the extent of ownership-dependent PSS modulation in bimanual TOJ. Interestingly, in a previous study by Moseley et al. (2008), participants could see not only the fake right hand but also their nonstroked left hand throughout the experiment. In such a setup, ownership of the visible real hand would remain unchanged. This methodological difference may affect the extent of the ownership-dependent PSS modulation.
